# The emerging role of guanine nucleotide exchange factors in ALS and other neurodegenerative diseases

**DOI:** 10.3389/fncel.2014.00282

**Published:** 2014-09-10

**Authors:** Cristian A. Droppelmann, Danae Campos-Melo, Kathryn Volkening, Michael J. Strong

**Affiliations:** ^1^Molecular Medicine Group, Robarts Research Institute, Western UniversityLondon, ON, Canada; ^2^Department of Clinical Neurological Sciences, Schulich School of Medicine & Dentistry, Western UniversityLondon, ON, Canada

**Keywords:** neurofilament, RNA binding proteins, GEF, neurodegeneration, motor neuron disease

## Abstract

Small GTPases participate in a broad range of cellular processes such as proliferation, differentiation, and migration. The exchange of GDP for GTP resulting in the activation of these GTPases is catalyzed by a group of enzymes called guanine nucleotide exchange factors (GEFs), of which two classes: Dbl-related exchange factors and the more recently described dedicator of cytokinesis proteins family exchange factors. Increasingly, deregulation of normal GEF activity or function has been associated with a broad range of disease states, including neurodegeneration and neurodevelopmental disorders. In this review, we examine this evidence with special emphasis on the novel role of Rho guanine nucleotide exchange factor (RGNEF/p190RhoGEF) in the pathogenesis of amyotrophic lateral sclerosis. RGNEF is the first neurodegeneration-linked GEF that regulates not only RhoA GTPase activation but also functions as an RNA binding protein that directly acts with low molecular weight neurofilament mRNA 3′ untranslated region to regulate its stability. This dual role for RGNEF, coupled with the increasing understanding of the key role for GEFs in modulating the GTPase function in cell survival suggests a prominent role for GEFs in mediating a critical balance between cytotoxicity and neuroprotection which, when disturbed, contributes to neuronal loss.

## INTRODUCTION

**FIGURE 1:**
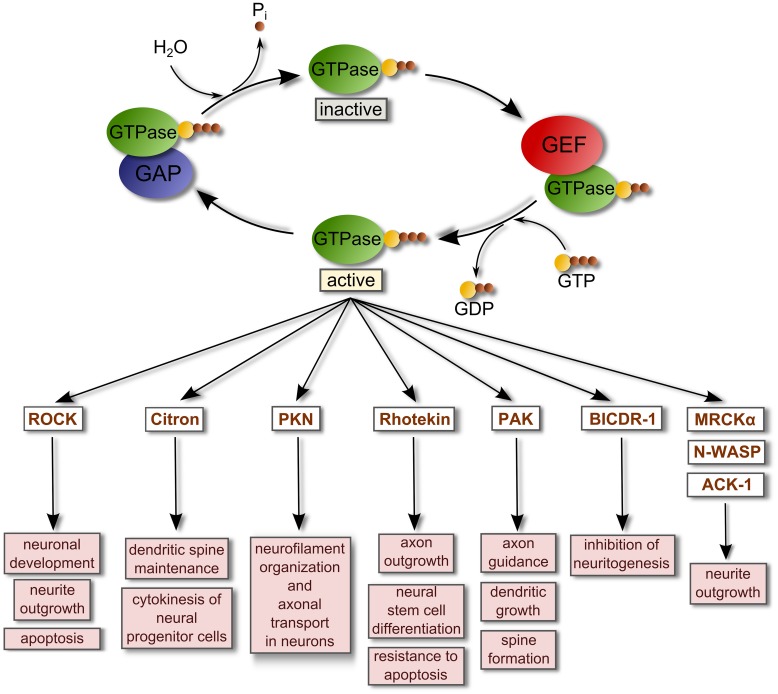
**Regulation of small GTPase activity by GEFs and GAPs.** Small GTPases are inactive when they are GDP-bound. GEFs bind and stabilize GDP-bound GTPases. However, due to the relatively high concentration of intracellular GTP these complexes rapidly dissociate into GTP-bound GTPases and free GEFs. When they are GTP-bound, GTPases regulate the activity of their binding targets or effectors to promote several cellular responses. GTPase-activating proteins (GAPs) stimulate the intrinsic hydrolytic capacity of Rho GTPases to promote GDP-bound forms, deactivating the GTPases and terminating the signaling. This reaction generates inorganic phosphate (P_i_). A list of representative effectors of active small GTPases related with the regulation of the nervous system is shown (Govek et al., 2005; Thumkeo et al., 2013; Villarroel-Campos et al., 2014). Abbreviations used are: ACK-1, Activated Cdc42-associated kinase 1; BICDR-1, Bicaudal-D-related protein 1; MRCKα, Myotonic dystrophy kinase-related Cdc42-binding kinase alpha; N-WASP, Neural Wiskott-Aldrich syndrome protein; PAK, P21 protein (Cdc42/Rac)-activated kinase; PKN, Serine-threonine protein kinase N; ROCK, Rho-associated coiled-coil containing protein kinase.

Small GTPases are low-molecular-weight (approximately 21 kDa) guanine nucleotide-binding proteins that function as binary molecular switches by alternating between an active GTP-bound state and an inactive GDP-bound state and in doing so, regulate the activation of several effectors (**Figure 1**). The five subfamilies of small GTPases (Ras, Rho, Rab, Sar1/Arf, and Ran) have highly diverse cellular roles. For example, GTPases of the Rho family (including Rho proteins, Rac proteins, Cdc42, TC10, TCL, Wrch1, Chp/Wrch2, and Rnd proteins; Van Aelst and D’Souza-Schorey, 1997; Burridge and Wennerberg, 2004) work through several effectors to influence a broad range of cellular processes, including cell cycle progression, vesicular trafficking, cell migration, intracellular actin dynamics, cell–cell interactions, response to cellular injury, axonal development, and guidance and neurite formation (Govek et al., 2005; Thumkeo et al., 2013). GTPases of the Rab family, which includes at least 66 different proteins (Klopper et al., 2012), function as regulators of specific intracellular traffic pathways, coordinating consecutive stages of transport, such as vesicle formation, vesicle and organelle motility, and tethering of vesicles to their target compartment (Zerial and McBride, 2001). Rab GTPases have important roles in neurons such as participating in synaptic vesicles fusion and endocytosis, axonal and dendritic transport, and neurite formation (D’Adamo et al., 2014; Villarroel-Campos et al., 2014).

The activation of small GTPases such as Rho or Rab proteins requires the participation of specialized enzymes called guanine nucleotide exchange factors (GEFs) which catalyze the exchange of GTP for GDP in the GTPase with the requirement of Mg^2+^. This event generates a GTP-bound active GTPase. Most GEFs belong to two broad families: the classical diffuse B-cell lymphoma (Dbl)-homology family in which the catalytic function is exerted by a tandem of two domains [a Dbl-homology domain (DH) and an adjacent pleckstrin-homology domain (PH) that together bind other proteins and phospholipids; Snyder et al., 2002; Rossman et al., 2005; Miller et al., 2014]; and, the dedicator of cytokinesis proteins (Dock) family of atypical GEFs that lack the Dbl domain. The highly conserved DH domain of the Dbl family of GEFs targets Dbl GEFs to the plasma membrane where binding to phospholipids is driven by the PH domain, while the DH domain interacts with inactive GTPase-GDP and catalyzes the exchange of GDP for GTP, activating the GTPase. Conversely, GTPase-activating proteins (GAPs) stimulate the intrinsic hydrolytic capacity of small GTPases to promote GDP-bound forms, deactivating the GTPases, and terminating signaling (Rossman et al., 2005; Lemmon, 2008; **Figure 1**).

Dbl GEFs are a highly complex family of about 80 proteins containing a variety of differing functional domains, including: (1) domains that modulate protein–protein interactions such as Proline rich domains (Ahn and Ye, 2005), PDZ binding domains (Garcia-Mata and Burridge, 2007), SH3 domains (van Rijssel and van Buul, 2012) and Fibronectin type-III domains (Rabiner et al., 2005); (2) modulators of GEF activity such as APC-binding region domains (Zhang et al., 2012) and Calponin homology domains (part of the actin binding domain superfamily; Yu et al., 2010); (3) modulators of plasma membrane interactions and localization such as FAK recognition domains (Zhai et al., 2003), CRAL-TRIO (SEC14) domains (van Rijssel and van Buul, 2012) and FERM domains (Kubo et al., 2002); and (4) modulators of protein structure/function such as Spectrin repeats (van Rijssel and van Buul, 2012), Zn binding domains (Gebbink et al., 1997), RGSL domains (Bielnicki et al., 2011), PDZ domains (Garcia-Mata and Burridge, 2007), and Leucine-rich domains (Gebbink et al., 1997). While perturbations in the Dbl GEF family have been best explored in cancer (Lazer and Katzav, 2011), they are also associated with a broad range of neurological disorders, including neurodegeneration.

**FIGURE 2:**
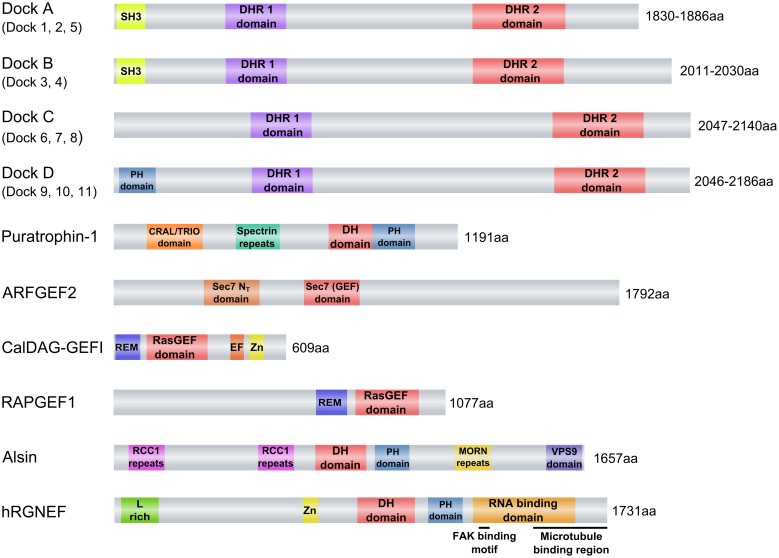
**Guanine nucleotide exchange factors (GEFs) associated with neurodegenerative diseases.** eIFB2 is not shown because its GEF catalytic activity is given by two subunits (epsilon and gamma) of a pentameric complex that forms the factor. Abbreviations used are: ARFGEF2, ADP-ribosylation factor GEF protein; C2 domain, C2 superfamily of C2 domain first identified in PKC; CalDAG-GEFI, calcium and diacylglycerol-regulated GEFI; CRAL/TRIO domain, SEC14 homology domain; DH domain, Dbl homology domain; DHR1, Dock Homology Region 1, a GEF domain; DHR2, Dock Homology Region 2, a GEF domain; Dock2, Dedicator of cytokinesis 2; Dock3, Dedicator of cytokinesis 3; EF, EF-hand motif (Calcium binding motif); hRGNEF, human Rho guanine nucleotide exchange factor; L-rich, Leucine-rich region; MORN repeats, Membrane Occupation and Recognition Nexus repeats; PH domain, Pleckstrin homology domain; RAPGEF1, Ras-related protein GEF1; RasGEF, Guanine nucleotide exchange factor for Ras-like small GTPases; RCC1 repeats, Regulator of chromosome condensation repeats; REM, Ras exchanger motif; Sec7, Domain named after the *S. cerevisiae* SEC7 gene product; Sec 7 N_T_, Amino-terminal Sec 7 domain; SH3, Src Homology 3 domain; Spectrin repeats, Spectrin-like repeats; Zn, cysteine-rich zinc binding domain. The schematics were based in the information obtained from the “conserved domain” search tool from NCBI (http://www.ncbi.nlm.nih.gov/Structure/cdd/wrpsb.cgi).

The Dock proteins are atypical GEFs in that they lack the DH-PH module and instead contain a Dock homology region (DHR)1-DHR2 module (**Figure 2**). This module functions similarly to the DH-PH module in that DHR1 mediates phospholipid binding and membrane targeting of Docks while DHR2 catalyzes nucleotide exchange through a mechanism distinct from the Dbl domain. The Dock proteins are further differentiated from the classical GEFs in that they exhibit specificity for activation of Rac and/or Cdc42, but not RhoA or other members of the Rho family. Eleven Dock proteins have been described in mammals (Dock1–11; **Table 1**). They are divided into four subfamilies based on sequence homology: Dock-A (includes Dock1/180, 2 and 5); Dock-B (includes Dock3, 4); Dock-C (also known as the zizimin-related or zir family, includes Dock6, 7, 8), and Dock-D (also known as the zizimin family, includes Dock9, 10, 11). Dock-A and -B members contain both a Src homology 3 (SH3) domain in the N-terminal region that can bind to the adaptor proteins ELMO (Engulfment and Motility) 1, 2, and 3 in addition to a proline-rich domain in C-terminus portion that binds Crk proteins. Dock-D members contain an N-terminal PH domain. Conversely, Dock-C members lack other recognizable domains outside of the DHR1–DHR2 module (**Figure 2**; Cote and Vuori, 2002; Meller et al., 2005). There is increasing evidence to suggest that perturbations in the function of both classical and atypical GEFs are associated with a broad range of disease states, including neurodevelopmental and neuropsychiatric disorders and neurodegeneration. Although neurodegeneration is a broad term generally used to describe a heterogeneous group of pathologies sharing the progressive loss of neuronal function and the death of neurons, one of their core neuropathological features is the presence of intra and extracellular protein aggregates (DiFiglia et al., 1997; Spillantini et al., 1997; Sieradzan et al., 1999; Goedert, 2001; Tiraboschi et al., 2004; Strong et al., 2005; Bloom, 2014). Classical examples of such neurodegenerative disorders include Alzheimer’s disease (AD), Parkinson’s disease (PD), Huntington’s disease (HD), and amyotrophic lateral sclerosis (ALS). In many of these disorders, GEF proteins are increasingly recognized as determinants of both cell survival and neurodegeneration. In this review, we examine the evidence for both classical and atypical GEFs in neurodegeneration, with a specific focus on the recently identified role of Rho guanine nucleotide exchange factor (RGNEF/p190RhoGEF) in the pathogenesis of ALS.

**Table 1:** Dock proteins, their role in the nervous system, and neurological disorders in which they have been implicated. Modified from Shi (2013) and Namekata et al. (2014).

Dock subfamily	Unique subfamily features	Preferential activation	Dock protein	Function	Related neurological diseases	Reference
Dock-A	Contains a N-terminal Src homology 3 (SH3) domain and a proline-rich C-terminus	Rac	Dock 1/180	Axon guidance, axonal pruning, dendritic spine morphogenesis, myoblast fusion	–	Li et al. (2008), Xu and Henkemeyer (2009), Kim et al. (2011)
			Dock 2	Lymphocyte migration; in the nervous system expressed exclusively in microglia and thus is linked to neuroinflammation	Alzheimer’s disease	Fukui et al. (2001), Cimino et al. (2009), Le Floc’h et al. (2013)
Dock-B	Contains a N-terminal Src homology 3 (SH3) domain and a proline-rich C-terminus	Rac	Dock3	Axonal growth and regeneration, neurite outgrowth, neuroprotection	Alzheimer’s disease, attention deficit hyperactivity disorder	Chen et al. (2001, 2005), de Silva et al. (2003), Namekata et al. (2013)
			Dock4	Establishment of axonal polarity, neurite differentiation, dendritic spine morphogenesis	Autism, dyslexia, schizophrenia	Ueda et al. (2008, 2013), Pagnamenta et al. (2010), Alkelai et al. (2012), Xiao et al. (2013)
			Dock 5	Myoblast fusion; mast cell degranulation	Parkinson disease (suggested association)	Laurin et al. (2008), Pankratz et al. (2011), Ogawa et al. (2014)
Dock-C	Contain only the DHR1–DHR2 module	Rac or Cdc42	Dock6	Neurite outgrowth, regulation of axonal growth and regeneration	–	Miyamoto et al. (2007, 2013)
			Dock7	Neuronal polarization, cortical neurogenesis, Schwann cell differentiation	–	Watabe-Uchida et al. (2006), Yamauchi et al. (2008, 2011), Yang et al. (2012)
		Cdc42	Dock8	T-cell and b-cell development; dendritic cell migration	Mental retardation, autism	Griggs et al. (2008), Randall et al. (2009), Harada et al. (2012)
Dock-D	Contain a N-terminal PH domain	Cdc42	Dock9	Dendrite development	Bipolar disorder	Detera-Wadleigh et al. (2007), Kuramoto et al. (2009)
			Dock10	Neurite dynamics	Autism	Nava et al. (2014)
			Dock 11	Lymphocyte migration	–	Sakabe et al. (2012)

## Dock PROTEIN FAMILY

The highly conserved dedicator of cytokinesis proteins (Dock) family of proteins play a key role in multiple aspects of neuronal development, including both axonal and dendritic differentiation, in addition to involvement in both neuroinflammation and the differentiation of Schwann cells (Watabe-Uchida et al., 2006; Miyamoto et al., 2007; Li et al., 2008; Yamauchi et al., 2008, 2011; Xu and Henkemeyer, 2009; Miyamoto and Yamauchi, 2010; Kim et al., 2011; Shi, 2013; Xiao et al., 2013). Not surprisingly therefore, Dock proteins have been increasingly recognized to be associated with a variety of neurodegenerative and neuropsychiatric disorders (**Table 1**). In Alzheimer’s disease, there is an increase in the number of Dock2-expressing microglia, a finding of pathogenic significance given that Dock2 deficiency reduces the size of β-amyloid (Aβ) plaque in cerebral cortex and hippocampus of a mouse model of AD (Cimino et al., 2009, 2013). It is likely that this effect is mediated through Dock2 association with the prostaglandin E2 receptor (EP2), which in turn regulates neuroinflammation (Liang et al., 2005; Cimino et al., 2009). Dock4, while also expressed in the lung, is highly expressed in the central nervous system (CNS) where it is concentrated in dendritic spines within the hippocampus during development and in adulthood (Ueda et al., 2013). It is associated with autism, dyslexia, and schizophrenia (**Table 1**; Pagnamenta et al., 2010; Alkelai et al., 2012). Less is known with respect to Dock10 and Dock11, although a rare Dock10 gene deletion is associated with autism spectrum disorders (Nava et al., 2014). Although an association between Dock5 and Parkinson’s disease has been suggested (Pankratz et al., 2011), the authors themselves noted that the Dock5 copy number variants were more likely to be variable numbers of tandem repeats as opposed to a typical copy number variation. Thus, the reports of Dock5 association with PD remain to be validated.

It is however Dock3 that has the greatest biological and therapeutic implications for neurodegenerative disorders, in part due to the fact that it is the only Dock protein that demonstrates high tissue specificity for the CNS. Initially discovered through a yeast two-hybrid assay as a binding partner to presenilin 1 that shares 40% homology with Dock1/180 [and termed presenilin binding protein (PBP); Kashiwa et al., 2000], PBP was found to be highly enriched in the CNS and to be specifically associated with Alzheimer’s associated pathology including neurofibrillary tangles, dystrophic neurites, and neuropil threads (Chen et al., 2001). PBP was subsequently renamed MOCA (“modifier of cell adhesion”) based on its ability to modulate cell-substratum adhesion and amyloid-β secretion (Chen et al., 2002). Based on its high sequence homology with Dock1/180 (51% homology with the SH3 domain; 45% homology with the DHR1 domain, 42% homology with the DHR2 domain, and the inclusion of a Crk binding site), Dock3/MOCA was identified as a member of the Dock180 superfamily of proteins (Cote and Vuori, 2002). Dock3/MOCA was initially thought to be devoid of GEF activity; however, it was subsequently shown to induce RAC-GTP loading through the interaction of its SH3 domain with ELMO1 (engulfment and cell motility protein 1), a mechanism shared with Dock1/180 (Grimsley et al., 2004; Namekata et al., 2004, 2012). Although the exact mechanism remains uncertain, PBP stimulates phosphorylation of tau at Ser199 (Chen et al., 2001) suggesting a direct mechanism in leading to the pathological hyper-phosphorylation of tau in Alzheimer’s disease. The interaction of Dock3 with the γ-secretase presenilin 1 (De Strooper et al., 2012) suggests a role in the Aβ precursor protein (APP) processing. This is further supported by the observation that Dock3 over-expression suppresses Aβ protein secretion (Chen et al., 2002). Dock3 also acts downstream of the APP-mediated signaling pathway and in doing so, promotes neuronal cell death (Tachi et al., 2012). Distinct from this role in the pathogenesis of Alzheimer’s disease, Dock3 has recently also been suggested to attenuate NMDA-mediated neurotoxicity and in doing so, prevent excitotoxic cell death (Bai et al., 2013; Namekata et al., 2013).

There is thus significant evidence to implicate the Dock family of GEFs in both neurodegeneration (largely restricted to Alzheimer’s disease) and a range of both developmental and neuropsychiatric disorders. As suggested earlier, this is not entirely unexpected given the integral role of GEFs in neural development.

## Dbl-HOMOLOGY GEFs ASSOCIATED WITH NEURODEGENERATION

While over 80 GEFs containing a Dbl-homology domain have been described, to date only a finite number have been associated with neurodegenerative disorders. The most lethal of these, presenting most often as a childhood disorder, is associated with mutations in any of the subunits of GEF eIF2B (eukaryotic initiation factor 2B). Conversely, alterations in the metabolism of the recently discovered Rho guanine nucleotide exchange factor (RGNEF or p190RhoGEF) are associated with the adult onset fatal disorder ALS. As is also typical of these proteins, the mechanisms by which these GEFs mediate neuronal degeneration is complicated by their pluripotential functions.

### eIF2B

The eukaryotic initiation factors (eIFs) play an essential role in the initiation of translation in eukaryotes by mediating the formation of a complex between Met-tRNA (initiator methionyl-tRNA), the AUG start codon of the mRNA and the 80S ribosome. Key to this process is eIF2, a classical G-protein which recruits Met-tRNA to the 40S ribosome when in its GTP-bound (active) form. Inactive GDP-bound eIF2 is regenerated by the GEF eIF2B in a process that is the major point of regulation of protein synthesis in all eukaryote cells (Mohammad-Qureshi et al., 2007). eIF2B consists of five subunits (α–ε), of which eIF2B ε constitutes the catalytic subunit which in turn is stimulated by the γ subunit (Anthony et al., 2000; Mohammad-Qureshi et al., 2007). Given the critical role of eIF2 in the initiation of translation, it is not surprising that the activity of eIF2B would be subject to tight regulation in order to regulate protein synthesis. Indeed, environmental stress leads to phosphorylation of eIF2 which impedes the recycling of eIF2 to the active GTP-bound form by actively competing with eIF2B, which in turn reduces translation globally (Wek et al., 2006; Jennings et al., 2013). This inhibition of translation initiation can also be driven by phosphorylation of eIF2B in response to environmental stress conditions, mediated through a small number of kinases including casein kinases I and II, and glycogen synthase kinase-3 (Pavitt et al., 1998; Welsh et al., 1998; Quevedo et al., 2000; Wang et al., 2001; Singh et al., 2006).

Mutations in the eIF2B ε-subunit were first described in patients with clinical and MRI characteristics of childhood ataxia with CNS hypomyelination (CACH)/leukoencephalopathy with vanishing white matter (VWM) neurodegenerative syndrome (Leegwater et al., 2001). Subsequently, mutations in the other four eIF2B subunits were identified in association with VWM disease (Leegwater et al., 2001; van der Knaap et al., 2002). This fatal inherited neurodegenerative disease is characterized mainly by progressive ataxia, spasticity, and variable optic atrophy, as well as seizures. While VWM disease is one of the most common childhood leukoencephalopathies, it can also be seen in later age groups (Ghezzi et al., 2012). Mutations in any of the eIF2B subunits can cause loss of GEF function of human and yeast eIF2B; some of them lead to almost complete loss-of-function (Fogli et al., 2004; Li et al., 2004; Richardson et al., 2004; Leng et al., 2011). Interestingly, the vast majority of the mutations found in eIF2B genes are missense mutations in the eIF2B ε-subunit (Fogli and Boespflug-Tanguy, 2006). Cell cultures from the brain of an individual with VWM carrying mutations in the eIF2B ε-subunit have shown that few GFAP-expressing astrocytes were present, induction of astrocytes was severely compromised and the few astrocytes generated showed abnormal morphologies and antigenic phenotypes. Consequently, it has been suggested that a deficiency in astrocyte function may contribute to the loss of white matter in VWM leukodystrophy (Dietrich et al., 2005).

### PURATROPHIN-1

Puratrophin-1 (Purkinje cell atrophy associated protein-1) is associated with autosomal dominant spinocerebellar ataxia (ADCA), a cerebellar pathology that manifests primarily as lack of motor coordination. This protein contains spectrin repeats, a GEF for Rho GTPases containing the classical Dbl homologous domain (Ishikawa et al., 2005) and a CRAL/TRIO domain (**Figure 2**). In fact, puratrophin-1 is a bona fide GEF that facilitates activation of the small GTPases Rac1, Cdc42, and RhoA. The overexpression of this protein induces rearrangements of the actin cytoskeleton, specifically enhancing the formation of lamellipodia and filopodia (Gupta et al., 2013). Mutations in the 5′ untranslated region of the PLEKHG4 (puratrophin-1) gene have been found in patients with ADCA. Interestingly, puratrophin-1 is aggregated in Purkinje cells of ADCA brains (Ishikawa et al., 2005).

### ARF GEF PROTEINS

The regulation of protein and lipid transfer within eukaryotic cells is largely mediated by vesicles that are formed in response to the need to transport between cellular compartments. The ADP-ribosylation factor (ARF) GEF family of proteins are important regulators of the initiation of vesicle formation in that they regulate the ARF GTPases through a GDP/GTP exchange (Donaldson and Jackson, 2011; Wright et al., 2014). The ARF GEF subfamily of proteins called the BIG/Sec7 ARF 1 GEFs activates ARF1. ARFGEF2 is one of two proteins in this family responsible for interior membrane trafficking in the trans-Golgi network and endosomes (Bui et al., 2009; **Figure 2**). ARFGEF2 has been postulated as a new potential biomarker of Huntington’s disease (HD) because of its significant up-regulation in blood samples of HD patients (Lovrecic et al., 2010).

### CalDAG-GEFI

Another guanine nucleotide exchange factor (GEF) that has been described as being involved in HD is a calcium and diacylglycerol-regulated GEFI (CalDAG-GEFI), also called RasGRP2 (**Figure 2**). CalDAG-GEFI is highly enriched in striatum and targets the small G proteins, Rap1 and Rap2 (Kawasaki et al., 1998; Ohba et al., 2000; Crittenden et al., 2004) and has been described to participate in the signaling pathway of M(1) muscarinic acetylcholine receptor (Guo et al., 2001). One of the most significant transcriptional changes of HD patients and animal models is the down-regulation of CalDAG-GEFI (Morton et al., 2005; Desplats et al., 2006, 2008; Kuhn et al., 2007). In a brain-slice explant model of HD, knock-down of CalDAG-GEFI expression rescues striatal neurons from pathology induced by transfection of polyglutamine-expanded Huntingtin protein (Htt) exon 1. This observation suggests that the striking down-regulation of CalDAG-GEFI in HD could be a protective mechanism that mitigates Htt-induced degeneration (Crittenden et al., 2010).

### RAPGEF1

The neuronal ceroid lipofuscinoses (NCLs) are the most common inherited neurodegenerative disorders mainly affecting children. That associated with mutations in the *CLN3* gene gives rise to CLN3 (ceroid-lipofuscinosis, neuronal 3) which manifests with a progressive retinopathy leading to blindness, dementia, epilepsy, and motor dysfunction. Microarray analyses have identified a GEF protein for small GTPase of the Ras family, Ras-Related Protein GEF1 (RAPGEF1 or C3G; **Figure 2**), which is dysregulated regardless of the clinical course of CLN3 disease, suggesting that this protein could be a potential biomarker (Lebrun et al., 2011). This is not surprising, considering the critical role of RAPGEF1 in multiple signal transduction pathways that regulate growth, differentiation, migration, and survival in neuronal cells (D’Arcangelo et al., 1995; Voss et al., 2006, 2008; Radha et al., 2008).

## Dbl-HOMOLOGY GEFs IN THE PATHOGENESIS OF ALS

Amyotrophic lateral sclerosis (ALS) is a neurodegenerative disorder characterized by the progressive loss of motor neurons leading to death within three to five years of symptom onset. While currently 1:350 men and 1:450 women are affected, increased incidence rates are predicted as the population ages (Strong, 2004; Murphy et al., 2008). Most cases are clinically sporadic ALS (sALS) with approximately 5% being familial (fALS; Byrne et al., 2011). The lack of treatments that fundamentally alter the disease course is in part related to the confounding array of disturbed cellular processes in ALS. However, there is increasing consensus that perturbations in RNA metabolism are critical to the disease process (Strong, 2010; Droppelmann et al., 2014).

One of the pathological hallmarks of ALS is the presence of neuronal cytoplasmic inclusions (NCIs) including RNA binding proteins such as mutant superoxide dismutase 1 (mtSOD1), TAR DNA binding protein (TDP-43), fused in sarcoma/translocated in liposarcoma (FUS/TLS), TATA-binding protein-associated factor 15 (TAF15), Ewing sarcoma breakpoint region 1 protein (EWS), and RNA binding motif protein 45 (RBM45; Stieber et al., 2000; Ge et al., 2005; Arai et al., 2006; Neumann et al., 2006; Kwiatkowski et al., 2009; Vance et al., 2009; Couthouis et al., 2011, 2012; Collins et al., 2012) in addition to NCIs consisting of intermediate filament cytoskeletal proteins such as neurofilament (NF; Kondo et al., 1986) and peripherin (He and Hays, 2004). The NF inclusions have been shown to arise in response to an alteration in the expression ratio of the three NF subunits (low, middle, and high molecular weight NFs; NFL, NFM, and NFH respectively, encoded by *NEFL*, *NEFM*, *NEFH*, respectively). The traditional view of NF function is that they are integral to the formation and maintenance of the cytoskeleton, including axon diameter and integrity. The contemporary view includes a critical role for NF as a protective sink for reactive nitrating species (Strong et al., 2003; Szaro and Strong, 2010), in cell signaling and transcriptional regulation (Eriksson et al., 2009), and in mitochondrial morphology, motility, and fusion (Gentil et al., 2012).

Notably, in ALS there is a selective loss of *NEFL* mRNA in spinal motor neurons while the levels of *NEFM* and *NEFH* mRNA are unchanged (Bergeron et al., 1994; Wong et al., 2000; Menzies et al., 2002). The biological importance of perturbing the expression ratio of NF has been repeatedly demonstrated both *in vivo* through the induction of selective motor neuron degeneration (Eyer and Peterson, 1994; Tu et al., 1997; McLean et al., 2005; Strong et al., 2005; Li et al., 2006) and *in vitro* (Lin et al., 2003, 2004, 2005; Strong et al., 2003). This evidence implies that alterations of *NEFL* mRNA levels on motor neurons could be associated with motor neuron death and consequently with ALS pathogenesis.

### ALSIN/ALS2

*ALS2* gene encodes for a protein called ALS2 or alsin. Alsin is a GEF protein predominantly expressed in central nervous system (Devon et al., 2005) that exhibits selective GEF activity on the members of small GTPase Rab5 (Rab5A, Rab5B, and Rab5C; Otomo et al., 2003; Topp et al., 2004). This protein has been involved in receptor trafficking, macropinocytic endocytosis, autophagosome-endolysosomal trafficking and axonal outgrowth (Devon et al., 2006; Hadano et al., 2006, 2010; Jacquier et al., 2006; Kunita et al., 2007; Otomo et al., 2008).

A comparison of alsin with other proteins reveals the presence of several interesting motifs. In its N-terminus domain, alsin contains a GEF domain for Ran GTPase, called RCC1. Alsin has been reported to have 5 RCC1 repeats that form a β-propeller similar to RCC1 protein in which this domain was first described (Dasso, 2001; Topp et al., 2004). The middle portion of alsin contains a tandem organization of diffuse B cell lymphoma Dbl homology (DH) and pleckstrin homology (PH) domains, the hallmark of GEFs for Rho-type GTPases (Rossman et al., 2005). The C-terminal region contains the vacuolar protein sorting 9 (VPS9) domain, which has been found in Rab5 GEFs (Zerial and McBride, 2001; Carney et al., 2006) and a tandem of eight membrane occupation and recognition nexus (MORN) motifs, which is implicated in the targeting and binding to the plasma membrane (Takeshima et al., 2000; Ma et al., 2006; **Figure 2**).

*ALS2* is a causative gene for the juvenile autosomal recessive form of multiple motor neuron diseases, including type 2 ALS (Hadano et al., 2001; Yang et al., 2001), primary lateral sclerosis (Yang et al., 2001) and infantile-onset ascending hereditary spastic paralysis (Eymard-Pierre et al., 2002). Several mutations distributed widely across the entire coding sequence of *ALS2* have been reported. They are predicted to result in either premature termination of translation or substitution of an evolutionarily conserved amino acid, leading to loss of its function (Hadano et al., 2001; Yang et al., 2001; Eymard-Pierre et al., 2002; Sztriha et al., 2008; Verschuuren-Bemelmans et al., 2008; Herzfeld et al., 2009; Mintchev et al., 2009; Shirakawa et al., 2009; Luigetti et al., 2013; Kocak Eker et al., 2014; Wakil et al., 2014). Despite all this evidence, studies using *ALS2* KO mice have demonstrated that the absence of alsin does not produce a severe phenotype. However, it has been reported that *ALS2* KO mice develop age-dependent deficits in motor coordination (Cai et al., 2005), an age-dependent and slow progressive loss of cerebellar Purkinje cells, a reduction in ventral motor axons during aging, astrogliosis, and evidence of deficits in endosome trafficking (Hadano et al., 2006), degeneration of corticospinal axons and axonal transport defects (Gros-Louis et al., 2008). In addition, primary cultured motor neurons lacking *ALS2* have been found to be more susceptible to oxidative stress (Cai et al., 2005).

### RGNEF/p190RhoGEF

RGNEF protein (Rho guanine nucleotide exchange factor) is a Rho-specific nucleotide exchange factor (GEF) that is part of the Dbl family of GEFs encoded by the *ARHGEF28* gene. This protein was first cloned from a mouse brain cDNA library and was named p190RhoGEF (Gebbink et al., 1997). Mouse RGNEF (mRGNEF) is ubiquitously expressed and it is able to specifically activate RhoA both *in vitro* and *in vivo* and binds to and co-localizes with microtubules (van Horck et al., 2001). mRGNEF can interact with c-Jun amino-terminal kinase (JNK) interacting protein-1 (JIP-1; Meyer et al., 1999), the 14-3-3 adapter protein (Zhai et al., 2001) and the Focal adhesion kinase (FAK; Zhai et al., 2003). The interaction with FAK is integral to the progression of colon carcinoma tumors (Yu et al., 2011).

Structurally, RGNEF has five important domains: an L-rich region and a cysteine-rich Zn binding domain in the amino terminus half of the protein; a Dbl homology domain (DH), a Pleckstrin homology domain (PH), and an RNA binding domain in the carboxyl terminus half of the protein (Gebbink et al., 1997; Canete-Soler et al., 2001; van Horck et al., 2001; Volkening et al., 2010; Droppelmann et al., 2013a; **Figure 2**). It is this latter aspect that renders RGNEF as being unique amongst the remaining members of the Dbl-homology GEF family of proteins in that it is the only member that combines RNA binding activity with GEF activity.

Mouse RGNEF can bind to a destabilizing element in the 3′ untranslated region (3′UTR) of the murine *NEFL* mRNA, consequently stabilizing the transcript (Canete-Soler et al., 1998, 2001). This interaction can be modulated by BC1 RNA (Ge et al., 2002), an untranslated 152-nucleotide polymerase III transcript that is highly expressed in large neurons of rat brain and spinal cord (DeChiara and Brosius, 1987). Interestingly, mRGNEF is involved in the NF protein aggregation formation observed in an RNA-triggered transgenic model of motor neuron disease (Nie et al., 2002; Lin et al., 2005) in which mRGNEF forms a protein complex with aldolase A and C which participates in the regulation of *NEFL* mRNA stability (Canete-Soler et al., 2005; Lin et al., 2005).

Given the evidence suggesting an important role for RGNEF in the regulation of RNA metabolism in motor neurons, we postulated that human RGNEF (hRGNEF) would play a key role in the pathogenesis of ALS. Our group determined that hRGNEF, similar to mRGNEF, is an RNA binding protein that regulates the stability of *NEFL* mRNA (Volkening et al., 2010; Droppelmann et al., 2013a). We observed that the RNA binding domain of hRGNEF could interact with *NEFL* mRNA in whole cell lysates (including proteins and RNA) from spinal cord in ALS but not control cases (Volkening et al., 2010). This observation was critical in that, in contrast to the effect of mRGNEF in stabilizing murine *NEFL* mRNA, we found that the effect of full length hRGNEF over human *NEFL* mRNA stability was the opposite. hRGNEF destabilizes *NEFL* mRNA and decreases the levels of NFL protein *in vitro* (Droppelmann et al., 2013a).

**FIGURE 3:**
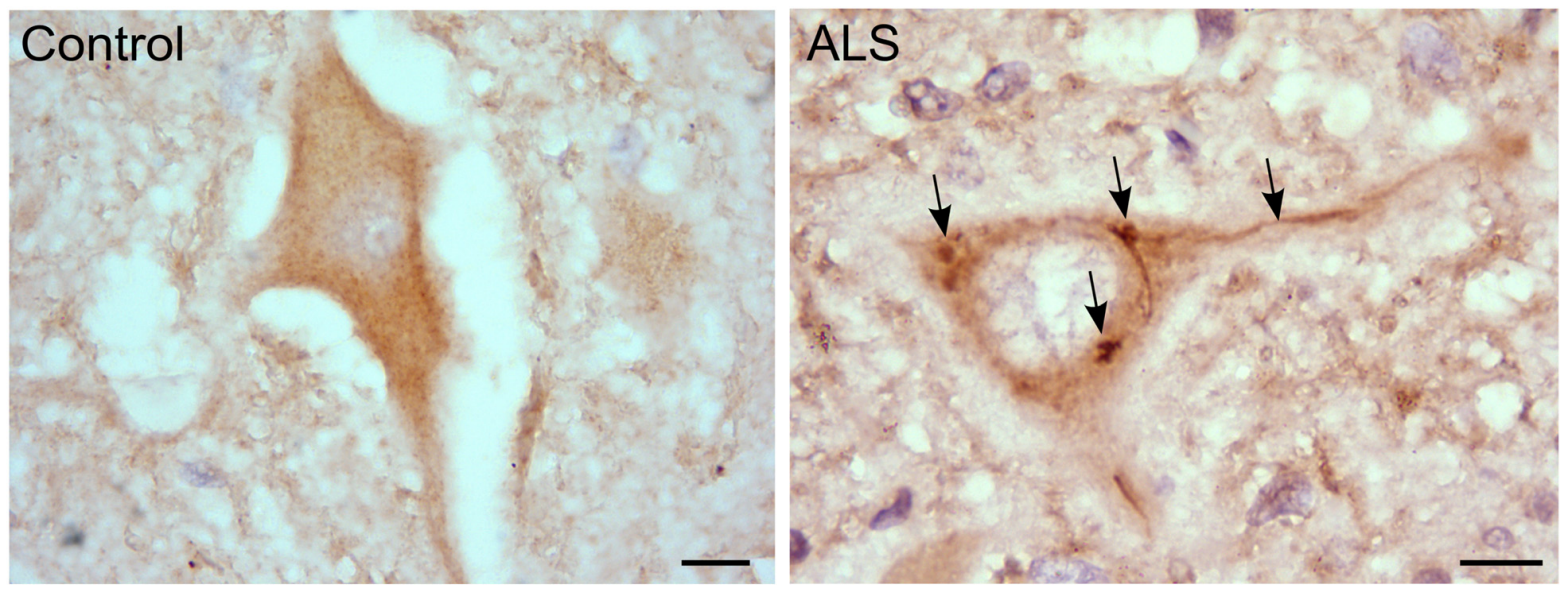
**RGNEF pathology in ALS motor neurons.** Representative image of RGNEF cytoplasmic inclusions observed in motor neurons of ALS patients (indicated by arrows) compared with a control case. Immunohisto chemistry was performed as described previously (Keller et al., 2012; Droppelmann et al., 2013a) using goat polyclonal anti-RGNEF (MediMabs; 1:500 dilution; Scale bar = 10 μm).

We also observed that hRGNEF immunoreactive NCIs in spinal motor neurons in ALS (**Figure 3**) co-localized with other RNA binding proteins, suggesting a common pathway of NCI formation in which multiple RNA binding proteins co-aggregate (Keller et al., 2012; Droppelmann et al., 2013a). Moreover, we and others found a heterozygous frameshift mutation in *ARHGEF28* in both fALS and sALS patients that predicts the expression of a truncated protein by the mutated allele (Droppelmann et al., 2013b; Ma et al., 2014).

It is interesting to note that mRGNEF has been described as an anti-apoptotic factor in neuronal cells (Wu et al., 2003). It was observed that mRGNEF confers protection against stress-induced apoptosis in neuronal cells which could be associated with the interaction of mRGNEF with JIP-1 and 14-3-3 proteins. This observation suggests that the sequestration of RGNEF into aggregates in ALS may contribute to neuronal death.

### C9ORF72

C9ORF72 is another gene strongly involved in neurodegeneration. An expanded GGGGCC hexanucleotide repeat in the first intron located between the 1st and 2nd non-coding exons of C9ORF72 is the most frequent genetic cause of frontotemporal dementia and ALS (DeJesus-Hernandez et al., 2011; Renton et al., 2011). C9ORF72 encodes a protein with largely unknown function. However, sensitive homology searches have shown that C9ORF72 is a full-length distant homolog of proteins related to Differentially Expressed in Normal and Neoplasia (DENN), a GEF that activates Rab-GTPases. These results suggest that C9ORF72 is likely to regulate membrane traffic in conjunction with Rab-GTPase switches (Levine et al., 2013).

## CONCLUDING REMARKS

There is compelling evidence for the emerging role of GEFs in neurodegeneration. Given the key role of many of these proteins in neuronal development and differentiation as well as cellular vesicular transport through the regulation of small GTPases, this is perhaps not unexpected. In the examples provided, the associated diseases range from lethal childhood onset disorders such a VWM disease, to developmental disorders such as the autism spectrum disorders, through to the fatal adult onset disorder ALS.

**FIGURE 4:**
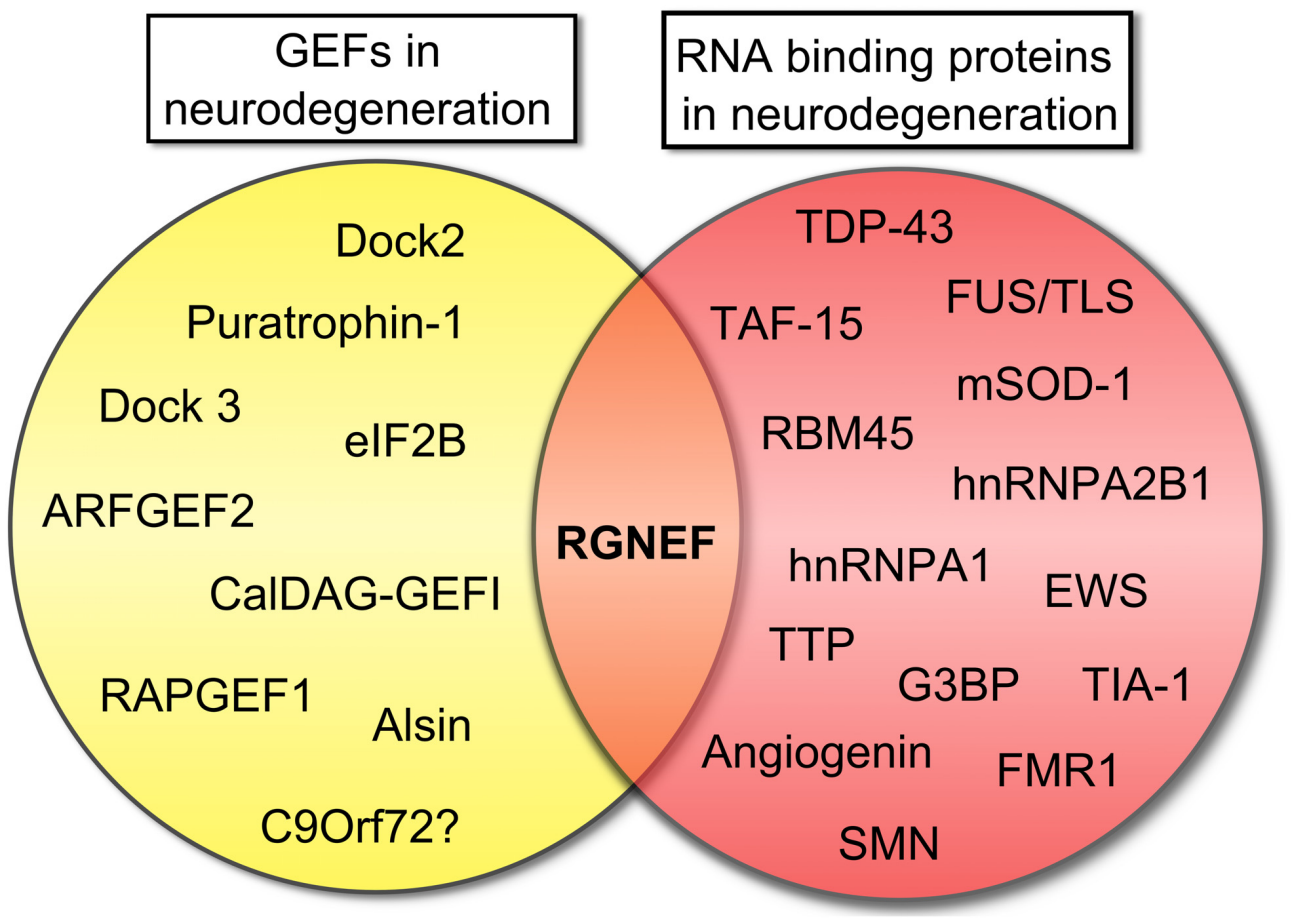
**Rho guanine nucleotide exchange factor (RGNEF) is the only GEF involved in neurodegeneration that also contains a RNA binding domain.** Scheme showing the participation of several GEFs in neurodegeneration as was described in this review and RNA binding proteins that participate in neurodegeneration (Ge et al., 2005; Vanderweyde et al., 2013). RGNEF is the only protein involved in neurodegeneration that can be grouped in both categories. Abbreviations are: ARFGEF2, ADP-ribosylation factor GEF protein; CalDAG-GEFI, calcium and diacylglycerol-regulated GEFI; Dock2, Dedicator of cytokinesis 2; Dock3, Dedicator of cytokinesis 3; eIF2B, eukaryotic initiation factor 2B; EWS, Ewing sarcoma breakpoint region 1 protein; FMR1, Fragile X mental retardation protein 1; FUS/TLS, Fused in sarcoma/Translocated in liposarcoma; G3BP, Ras-GTPase-activating protein SH3-domain-binding protein; hnRNPA1, heterogeneous nuclear ribonucleoprotein A1; hnRNPA2B1, heterogeneous nuclear ribonucleoprotein A2/B1; mSOD1, mutant superoxide dismutase 1; RAPGEF1, Ras-related protein GEF1; RBM45, RNA binding motif protein 45; RGNEF, Rho guanine nucleotide exchange factor; SMN, Survival motor neuron protein; TAF15, TATA-binding protein-associated Factor 15; TDP-43, TAR DNA binding protein; TIA-1, T-cell intracellular antigen 1; TTP, Tristetraprolin.

In this context, the example of RGNEF and its association with ALS is notable as this is the only GEF that combines RNA binding activity with the capacity to activate RhoA GTPase (**Figure 4**). The observation that RGNEF is able to immunoprecipitate *NEFL* mRNA from ALS spinal cord homogenates and not from controls suggests a preferential interaction in ALS that would be predicted to suppress *NEFL* mRNA levels, consistent with the loss of *NEFL* mRNA detected by *in situ* hybridization in ALS motor neurons (Wong et al., 2000). This may be further augmented through the sequestration of multiple RNA binding proteins within pathological NCIs in ALS spinal motor neurons with the net effect being a loss of the regulation of *NEFL* and other mRNAs stability. In a similar vein, the sequestration of RGNEF within NCIs in ALS would also be predicted to render it unavailable to participate in the modulation of RhoA GTPase activation and potentially lead to neuronal death. Understanding this intricate relationship is currently the focus of ongoing studies.

It is also interesting to postulate that alterations in GEF activity, while not directly inducing a disease state, could function as a critical “second insult” through the loss of one or more effector pathways. For instance, while the loss of *NEFL* mRNA stability is critical to the formation of pathological NCIs in spinal motor neurons, the concomitant loss of GEF activity due to RGNEF sequestration may be sufficient to render the cell incapable of responding to the subsequent cellular stressors. Such a conceptualization is consistent with the increasing evidence for oligogenic inheritance in ALS where a second gene might participate as a critical modulator of the pathological phenotype of the disease (van Blitterswijk et al., 2012; Droppelmann et al., 2013b). In this context, perturbations in the function of GEF proteins might well be considered as key candidates for this “second hit” because of their direct or indirect roles on cell survival (Chen et al., 2002, 2009; Wu et al., 2003; Cai et al., 2005; Dietrich et al., 2005; Crittenden et al., 2010; Bai et al., 2013; Namekata et al., 2013).

## Conflict of Interest Statement

The authors declare that the research was conducted in the absence of any commercial or financial relationships that could be construed as a potential conflict of interest.
